# *In silico* Design of Laccase Thermostable Mutants From Lacc 6 of Pleurotus Ostreatus

**DOI:** 10.3389/fmicb.2018.02743

**Published:** 2018-11-14

**Authors:** Rubén Díaz, Gerardo Díaz-Godínez, Miguel Angel Anducho-Reyes, Yuridia Mercado-Flores, Leonardo David Herrera-Zúñiga

**Affiliations:** ^1^Laboratory of Biotechnology, Research Center for Biological Sciences, Autonomous University of Tlaxcala, Tlaxcala, Mexico; ^2^Agrobiotechnology Laboratory, Polytechnic University of Pachuca, Hidalgo, Mexico; ^3^Division of Environmental Engineering Technology of Higher Studies of East Mexico State, Mexico City, Mexico; ^4^Area of Biophysical Chemistry, Department of Chemistry, Metropolitan Autonomous University-Iztapalapa, Mexico City, Mexico

**Keywords:** laccase, *Pleurotus ostreatus*, Lacc 6, mutants, energy minimization

## Abstract

Fungal laccase enzymes have a great biotechnological potential for bioremediation processes due to their ability to degrade compounds such as ρ-diphenol, aminophenols, polyphenols, polyamines, and aryldiamines. These enzymes have activity at different pH and temperature values, however, high temperatures can cause partial or total loss of enzymatic activity, so it is appropriate to do research to modify their secondary and/or tertiary structure to make them more resistant to extreme temperature conditions. *In silico*, a structure of the Lacc 6 enzyme of *Pleurotus ostreatus* was constructed using a laccase of *Trametes versicolor* as a template. From this structure, 16 mutants with possible resistance at high temperature due to ionic interactions, salt bridges and disulfide bonds were also obtained *in silico*. It was determined that 12 mutants called 4-DB, 3-DB, D233C-T310C, F468P, 3-SB, L132T, N79D, N372D, P203C, P203V, T147E, and W85F, presented the lowest thermodynamic energy. Based on the previous criterion and determining the least flexibility in the protein structures, three mutants (4-DB, 3-DB, and P203C) were selected, which may present high stability at high temperatures without affecting their active site. The obtained results allow the understanding of the molecular base that increase the structural stability of the enzyme Lacc 6 of *Pleurotus ostreatus*, achieving the *in silico* generation of mutants, which could have activity at high temperatures.

## Introduction

One of the greatest qualities studied and demanded by protein engineering in the environmental and biotechnological fields is the search for conformational and structural stability of proteins when subjected to high temperatures, due to the fact that a high percentage of industrial processes are carried out in such conditions.

The native structure of a protein is stabilized by physicochemical interactions (Djikaev and Ruckenstein, 2008; Das et al., 2018); nevertheless, the free energy between the folded and unfolded states has been estimated in an order of 5–20 kcal/mol, which would be equivalent to breaking a small number of such interactions (Venkataramani et al., 2013; Nawaz et al., 2015; Dubey et al., 2017), in this way, structural stability turns out to be a delicate balance of interactions. Proteins can lose their stability and be denatured by various physicochemical treatments such as: heat, pressure, pH, urea or guanidine chloride, among others (Govardhan, 1999). When the bonds that maintain the native structure of a protein are broken, most of the time it loses its functionality due to structural destabilization. One of the processes of protein denaturation most studied is by heat, which occurs in a relatively narrow temperature range. That is why there are multiple proposals to increase their structural resistance at high temperatures (Gao et al., 2016; Kumar et al., 2016; Kean et al., 2017). An example is the hydrophobic packaging where the amino acid residues are oriented toward the protein core, being the main stability factors (Reed et al., 2013; Luna-Martínez et al., 2016); It should be mentioned that studies on directed mutations carried out for the filling of hydrophobic cavities have resulted in an improvement of stability by hydrophobic packaging (Christensen and Kepp, 2013a; Oda and Kinoshita, 2015). Likewise, electrostatic interactions that occur between amino acids of opposite charge, also have great importance to maintain the stability of the conformation of the protein (Sohini and Srikanta, 2009; Christensen and Kepp, 2013a).

In studies on protein flexibility, it has been found that mutations through which conformational degrees of freedom are reduced confer structural resistance to proteins (Zhou et al., 2008; Gribenko et al., 2009). A type of covalent bond that allows the reduction of flexibility is formed by the union of two cysteines, called disulfide bridge and is considered as a primary factor for stability (Radestock and Gohlke, 2011). However, the success in stabilization by disulfide bridges in protein engineering is dependent on the conformational change caused by the themselves folding and the effects of substituting the native residues with cysteines (Pappenberger et al., 1997). Other tactics to reduce flexibility is the substitution of free chain amino acids for prolines and the shortening in the length of loops, which can increase the stability of proteins by compaction. Although today part of the knowledge about flexibility, mobility and protein dynamics are derived from experimental data, it is true that currently there is no experimental technique to monitor conformational changes at the molecular level and atomic resolution as a function of time. For this reason, the molecular, conformational, flexibility and mobility details are often studied by computational simulation techniques providing a possibility to obtain dynamic information on the functionality of proteins (Anbar et al., 2012; Yakimov et al., 2016).

On the other hand, the laccase enzymes (EC.1.10.3.2) that belong to the family of multicopper-oxidases, can oxidize numerous compounds with an oxide-reduction potential that varies between 500 and 800 mV, such as the ρ- diphenol, aminophenols, polyphenols, polyamines and aryl diamines (Xu, 2005). They are N-glycosylated proteins (10–45% of their molecular mass), in general, they are in monomeric form with molecular weight of 20–80 kDa, their pI is between 2.6 and 4.5, being biologically active at pH 2.0–8.5 (Wesenberg et al., 2003; Díaz et al., 2013). These enzymes have been described in bacteria, fungi, plants and insects and more than 100 different proteins have been identified (Shuresh-Kumar et al., 2003). Its physiological function in plants is in the lignification process and in fungi in morphogenesis processes (formation of spores, pigments of fruiting bodies), pathogenesis, virulence and degradation of lignin, but it has also been reported that in some insects act in the formation of cuticle in the process of sclerotization (Kramer et al., 2001) and in bacteria have the function of monomer cross-linking, polymer degradation and the breakdown of aromatic rings (Sharma et al., 2007). The laccases have a great biotechnological potential, for example in the whitening of paper pulp, in the stabilization of wines and beers, in the food industry, in the cosmetic and pharmaceutical industries and in various bioremediation processes including discoloration of textile dyes and degradation of xenobiotic compounds (Mayer and Staples, 2002).

The laccase enzyme Lacc 6 produced by *Pleurotus ostreatus* has shown a high level of expression under different development conditions and has been partially characterized, determining its molecular weight, pI, optimum pH of activity, Km on different substrates, etc. (Baldrian, 2006; Díaz et al., 2013), because of the above, it was considered to be modified through protein engineering obtaining *in silico* mutants with greater thermodynamic stability, for its possible application in the degradation of polluting compounds of phenolic origin and recalcitrants that are in extreme temperature conditions.

In this research, a structure of the Lacc 6 enzyme of *Pleurotus ostreatus* was constructed *in silico*, using a laccase of *Trametes versicolor* as a template. From this structure, 16 mutants with possible resistance at high temperature due to ionic interactions, salt bridges and disulfide bonds were also obtained *in silico*.

## Materials and Methods

### Modeling of Lacc 6 of *Pleurotus ostreatus*

The initial alignment was made using ClustalO^1^ and the secondary structure prediction with Predictprotein program^2^. The template structure to Lacc 6 model with the highest degree of homology, was search (non-redundant) in BLAST server (Basic Local Alignment Search Tool) through PDB^3^ (Boratyn et al., 2012). The structural model of the native Lacc 6 laccase from *Pleurotus ostreatus* was carried out using the Modeller computer package^4^ (Webb and Sali, 2016).

### Preparation of Mutant Proteins of Lacc 6

The thermostable Lacc 6 quimeric protein was determinated by two ways: 1) making an exhaustive literature search to find all laccases with t1/2 over 55°C (Hildén et al., 2009) to structure multiple models of them whith Modweb-ModBase server^5^ to find the invariant structural motif-interaccions, and 2) finding the most favorable and thermodynamic residue shift in Lacc 6 by PopMusic server^6^ (Dehouck et al., 2011), each of any shitf were constructed using UCSF CHIMERA^7^ with SWAPAA algorithm that performs a side chain replacement by rotamers search selecting the lowest score and shocks between the new side chain and chains of its neighborhood (Fersht et al., 1986).

### Minimization of Thermodynamic Energy of Lacc 6 Mutants

Once the mutants were built, they were minimized by the steepest descent protocol up to 0.01 kcal/mol, with the NAMD 2.12 package (Scalable Molecular Dynamics) (Phillips et al., 2005), using the CHARMM36 (Chemistry at Harvard Macromolecular Mechanics) force field (Huang et al., 2017), it was performed with explicit solvent at a physiological sodium chloride (NaCl) ion concentration of 0.15 M, the parameters for the copper atoms were taken from Ungar et al. (1997).

### Normal Mode Analysis

After the minimization, the structures with the lowest energy, these assumed as the most stable energetically (thermostable mutants) (Burkoff et al., 2012), to later be studied through by the Normal Modes theory with the elNémo server, available at http://www.sciences.univ-nantes.fr/elnemo/ (Suhre and Sanejouand, 2004). The thermostable mutants were always contrasted with Lacc 6 throughout all the work, and all images were constructed using Visual Molecular Dynamics (VMD) Sofware (Humphrey et al., 1996).

## Results

The Lacc 6 sequence of *Pleurotus ostreatus* shown below (UniProtKB: A0A067NQH1_PLEOS), which is constituted by 533 amino acids, where the first 21 residues correspond to the signal peptide.

**FIGURE 1:**
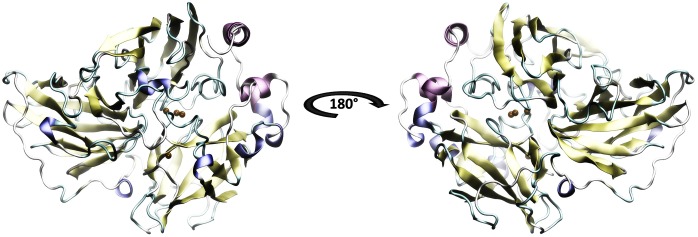
Model of the laccase enzyme of *Pleurotus ostreatus*. In color-coded the secondary structure is presented: yellow, beta sheets; purple and blue, alpha helix; in green and white, loops and non-structured areas. In ocher color the copper atoms belonging to the active site are represented. The 180 degrees rotation allows to appreciate the three structural domains which are defined by Domain A of amino acids 1–131, Domain B of amino acids 132–301 and Domain C the last 194 amino acids.

The secondary structure prediction of Lacc 6 was performed in Predictprotein program^8^ (Yachdav et al., 2014). The initial alignment was performed using 142 laccase sequences from *Trametes* using the ClustalO program^8^ (Sievers et al., 2011) and the best homologous structure obtained with the BLAST server was the laccase of *Trametes versicolor* 1GYC (Protein Data Bank, PBD), it is worth mentioning that the alignment was consistent with the template structure and the active sites were perfectly aligned between them. Consequently, 1GYC structure was used as a template to make the model of Lacc 6, the homology between its sequence and 1GYC was close to 62% (UniProt Consortium 2012; Coluzza, 2017). The model of Lacc 6 (Figure 1) was built through Modeller computer package with a DOPE score of −1.88. On the other hand, the Lacc 6 model and the prediction of its secondary structure were highly related. The motifs of the Lacc 6 model were characterized by the PDBsum program, by which it was determined that the scaffolding of Lacc 6 consists of: 7 β-sheets, 9 β-hairpin, 8 β-bulge, 28 β-sheets, 10 α-helices (2 of them with helix-helix interactions), 44 β-turns, 8 γ-turns, and 2 disulfide bridges. Once the model was constructed and verified, it was proceeded to be minimized with the purpose of eliminating the steric shocks.

To generate a thermostable mutant of Lacc 6 according to structural evolution, an exhaustive search of literature on laccases with thermal resistance was carried out (Pezeshgi-Modarres et al., 2016; Coluzza, 2017), of which almost all those found were modeled and reported in Table 1.

**Table:** 

10	20	30	40	50
MAVAFVALVS	LALALVRVEA	SIGPRGTLNI	ANKVIQPDGF	SRSTVLAGGS
60	70	80	90	100
YPGPLIKGKT	GDRFQINVVN	KLADTSMPVD	TSIHWHGLFV	KGHNWADGPA
110	120	130	140	150
MVTQCPIVPG	HSFLYDFEVP	DQAGTFWYHS	HLGTQYCDGL	RGPLVVYSKN
160	170	180	190	200
DPHKRLYDVD	DESTVLTVGD	WYHAPSLSLT	GVPHPDSTLF	NGLGRSLNGP
210	220	230	240	250
ASPLYVMNVV	KGKRYRIRLI	NTSCDSNYQF	SIDGHTFTVI	EADGENTQPL
260	270	280	290	300
QVDQVQIFAG	QRYSLVLNAN	QAVGNYWIRA	NPNSGDPGFE	NQMNSAILRY
310	320	330	340	350
KGARSIDPTT	PEQNATNPLR	EYNLRPLIKK	PAPGKPFPGG	ADHNINLNFA
360	370	380	390	400
FDPATALFTA	NNHTFVPPTV	PVLLQILSGT	RDAHDLAPAG	SIYDIKLGDV
410	420	430	440	450
VEITMPALVF	AGPHPLHLHG	HTFAVVRSAG	SSTYNYENPV	RRDVVSIGDD
460	470	480	490	500
PTDNVTIRFV	ADNAGPWFLH	CHIDWHLDLG	FAVVFAEGVN	QTAAANPVPE
510	520	530		
AWNNLCPIYN	SSNPSKLLMG	TNAIGRLPAP	LKA


**Table 1:** Catalytic properties at high temperatures of laccase enzymes and accessions data.

Organism	t_opt(°C)_	t_1/2(°C)_	t_1/2(°h)_	UniPropt-Entry	PDB ID or Template	% Homology	Reference
*Albatrella dispansus*	70	nr	nr		–	–	Wang and Ng, 2004
*Basidiomycete PM1 (CECT 2971)*	80	nr	nr	Q12571	5ANH	–	Coll et al., 1993
*Coprinus cinereus*	60–70	60	0.5	Q9Y780	1HFU	–	Schneider et al., 1999
*Coriolopsis gallica* (A-241)	70	60	nr	Q9P8G4	1GYC 3KW7 5NQ8	65 65 65	Calvo et al., 1998
*Daedalea quercina* (CCBAS528)	70	65	0.5	Q6VPS6	1GYC 2XYB 5NQ8	74 67 68	Dedeyan et al., 2000
*Marasmius quercophilus*	75–80	40	48	AF162785	5ANH 2HRG 5A7E	99 97 96	Baldrian, 2006
*Physisporinus rivulosus*	nr	70	1.0	I1W1V7	3KW7 5NQ7 2XYB	73 70 70	Hildén et al., 2007
*Steccherinum ochraceum*	70	70	1.7	I1SB14	5ANH	–	Chernykh et al., 2008
*Trametes gallica*	70	nr	nr	C5IXN8	3KW7 5A7E 4A2D	73 71 71	Dong and Zhang, 2004
*Trametes versicolor*	80	nr	nr	Q5IR80	1GYC 3X1B 5LDU	99 77 80	Koschorreck et al., 2008
*Tricholoma giganteum*	70	nr	nr		–	–	Wang and Ng, 2004
*Peniophora sp. UD4*	70	nr	nr	A0A2I6HE30	2QT6 4JHU 4A2D	63 63 62	Jordaan and Leukes, 2003
*Melanocarpus albomyces*	60–70	60	5.0	Q70KY3	2Q9O	–	Kiiskinen et al., 2004
*Myceliophthora thermophila*	nr	70	0.3	G2QG31	1GW0 3PPS 3SQR	75 68 37	Berka et al., 1997

*t_opt(°C)_: Optime temperature, t_1/2(°C)_: Media temperature, t_1/2(h)_: Media time, nr: Not reported.*

Once discovered the thermostable laccases and built their models, this type of proteins was studied with the purpose to find their structural motifs invariants such as salt bridges, ionic interactions and disulfide bonds (Fersht et al., 1986). The structural invariant interactions between Lacc 6 and models or structures of thermostable laccases can be see in Table 2. Location of each mutation was obtained by Lacc 6 model overlap whit any thermostable laccases. Were determined structurally the networks of conserved electrostatic interactions of thermal laccases (Table 2, Columns 2 and 3).

**Table 2:** Mutants with potential to confer thermal stability or disturbance the active site of the enzyme Lacc 6.

	Alignment	Disulfide bridges	Ionic interactions	Disturbance of active site
Mutant	S431D	D233C-T310C	G234R	F468P
	I217F	H184C-S284C	S264E	F481M
	V79D	K396C-D399C	T147E	G420L
	N275K	N94C-G465C	G234K/Q271E/N270D (*3-SB*)	L132T
	N275R	D233C-T310C/H184C-S284C	G234E/Q271K/N270R	L416I
	V372D	D233C-T310C/H184C-S284C/ K396C-D399C (*4-DB*)	G234D/Q271K/N270R	L469F
	Q65R	D233C-T310C/H184C-S284C/K396C-D399C/N94C-G465C	G234R/Q271E/N270D (*3-SB*)	W85V
				S130D
				W85F

*Second column shows the mutations obtained by multiple alignment, these residues are highly conserved over time; in columns three to five, the mutants were build through structure-overlapping between Lacc 6 and the all other models/structures to find the conservative structures motifs. Disulfide bridges and Ionics interaccions are deposited in three and four columns, respectively. In the last column are the mutants that can break the active site.*

By another way, the PoPMuSic hot points (most favorable thermodynamic residue shift) with the ability to confer structural stability to Lacc 6 at high temperatures can be see in Table 3.

**Table 3:** Main mutations obtained by PoPMuSiC program.

	Hot points	Shift	ΔΔG (kcal/mol)
1	203	PRO → VAL	−1.91
2	203	PRO → CYS	−1.89
3	487	GLU → ILE	−1.75
5	487	GLU → TYR	−1.75
6	170	ASP → TYR	−1.5
7	170	ASP → PHE	−1.47
8	399	ASP → MET	−1.37
9	142	GLY → PHE	−1.35
10	183	PRO → TRP	−1.2

*The 10 mutants (shift residue) with the best and favorable energy in contrast to Lacc 6. The amino acid sequence number is found in second colum, in the next columna is the hot poin residue and their shift, the last column presnt the ΔΔG between Lacc 6 and mutant protein.*

**FIGURE 2:**
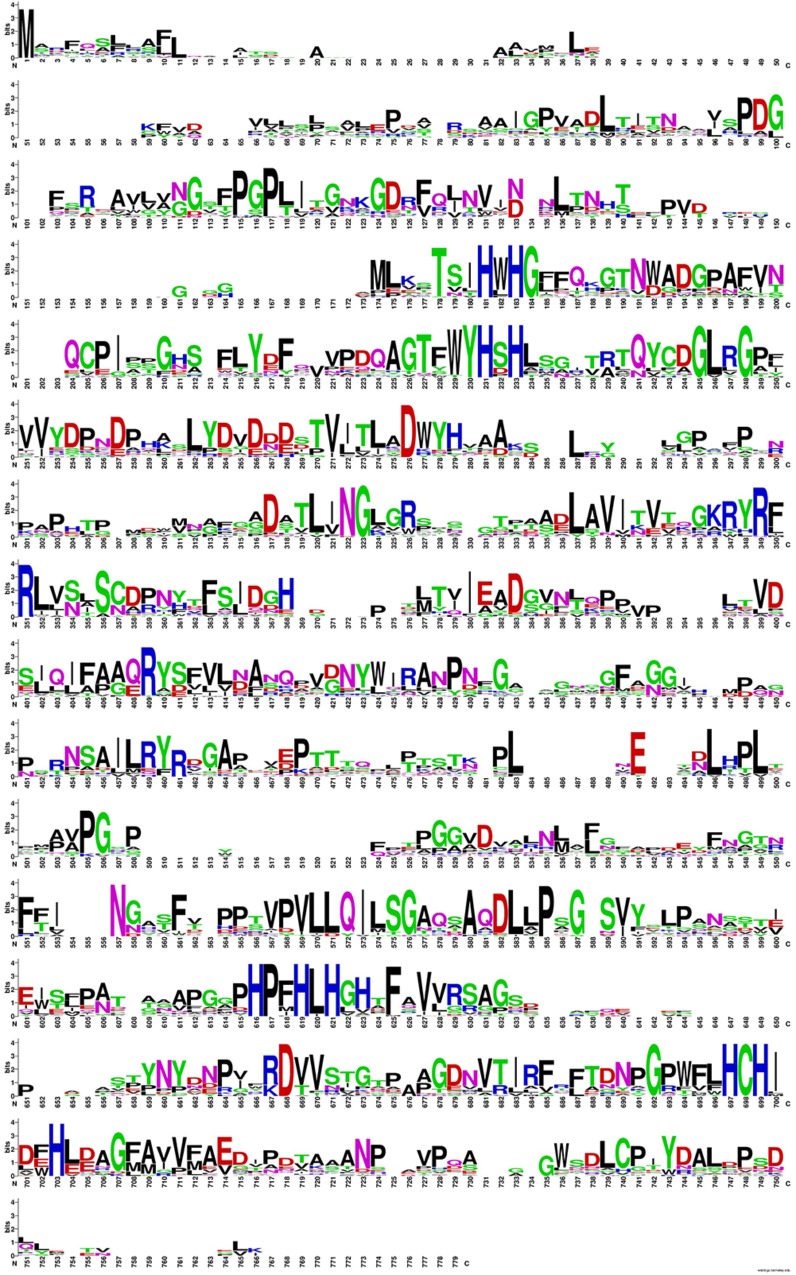
Propensity diagram for the alignment of the 16 thermoresistant sequences. In blue color the conservation of Histidines that conform the active site for this class of enzyme is shown, the positions of greater conservation (greater amplitude) generate clues of evolutionary invariants and important for the structural stability of these enzymes.

Table 3 contains the shift residue information, the evolutionary invariants with high capacity of confer thermoresistant in Lacc 6 enzyme sequences are reflected on this. The alignment (Figure 2) of the 16 structures, created by WebLogo program^10^, capture the conservation propensity of each mutate residue set in column 3, with this approach we can see that the best single mutant is P203V showing a ΔΔG = −1.91 kcal/mol below the Lacc 6.

Based on the Tables 2, 3, a mutant energy minimization was performed in each one, to define the most suitable shift that could confer structural resistance to Lacc 6 (Liwo et al., 2002; Nakagawa and Peyrard, 2006). The mutant energy ponderation on the Table 4 shows that 4-DB, 3-DB, and D233C-T310C (highlighted in gray), are the most favorable energy mutants with the ability to confer structural thermal-resistance. In this singular way, was appreciate that the best designed laccases by the evolution are those with a disulfide bridges networks such as the mutants mentioned above.

**Table 4:** Energy difference between the native enzyme and their mutants.

Method	Mutant	ΔE (kcal/mol)
PoPMuSiC	D170F	0.067
	D170T	0.063
	D399M	0.009
	E487I	0.040
	E487Y	0.091
	G142F	0.066
	P183T	0.094
	P203C	−0.063
	P203V	−0.025
Alignment	G431D	0.013
	I217F	0.020
	N275K	0.068
	N275R	0.045
	N372D	−0.003
	N79D	−0.050
	Q65R	0.007
Ionic interaction	G234D-N271K-N270R	0.073
	G234E-N271K-N270R	0.059
	G234k-N271E-N270D	−0.010
	G234R	0.184
	(*3-SB*) G234R-N271E-N270D	−0.094
	S264E	0.038
	T147E	−0.044
Disulfide bridges	D233C-T310C/H184C-S284C	−0.002
	(*3-DB*) D233C-T310C/H184C-S284C/K396C-D399C	−0.131
	(*4-DB*) D233C-T310C/H184C-S284C/K396C-D399C/N94C-G465C	−0.177
	D233C-T310C	−0.100
	H184C-S284C	−0.064
	K396C-D399C	−0.037
	N94C-G465C	0.007
Disturbance of active site	F468P	−0.091
	F481M	−0.022
	G420L	0.136
	L132T	−0.084
	L416I	0.029
	L469F	−0.009
	M85V	0.014
	S130D	−0.017
	W85F	−0.042

*In the first column are represented the search method for the proposed mutants; second column, the proposed residue shift for the mutants design and the last column presents the mutant energy after the minimization, taking as a baseline the Lacc 6 energy.*

**FIGURE 3:**
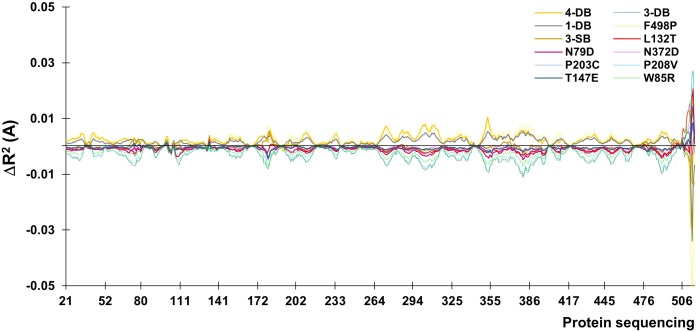
Values of mean displacement for the difference between the mutated enzyme and its native counterpart.

Once the energy differences were quantified and analyzed, was continued with a Normal Modes study of the thermostable mutants (4-DB, 3-DB, D233C-T310C, 3-SB, F468P, L132T, and others). The intention of Normal Modes Study is know the collective and correlated movements of the lower energy mutant modes (Tama and Sanejouand, 2001). The modes emitted by the ElNemo program include six trivial zero order frequencies. This study focused on mode 7, corresponds to the protein internal movements identified in direction of maximum movement; 10 snapshots of any structure (referring to the minimized initial structure) were obtained to display and represent the mode dynamics. The difference in the mean squared value of the normalized displacement (R^2^) of each alpha carbon (Cα) is plotted as a function of the number of residues, where for all residues, significantly mobile regions are seen above and still below the line in the graph of Figure 3.

The movements of the Cα for normal mode 7 are plotted as a function of the number of residues. The residue with the least displacement appears with a minimum value of *R*^2^ below the line at the origin. The higher amplitude values are due to the zones near to the cavity and to the terminal helix, the latter usually being a meaningless section since it is usually an artifact of the software itself, it is usually eliminated before calculations (Altschul et al., 1990).

**FIGURE 4:**
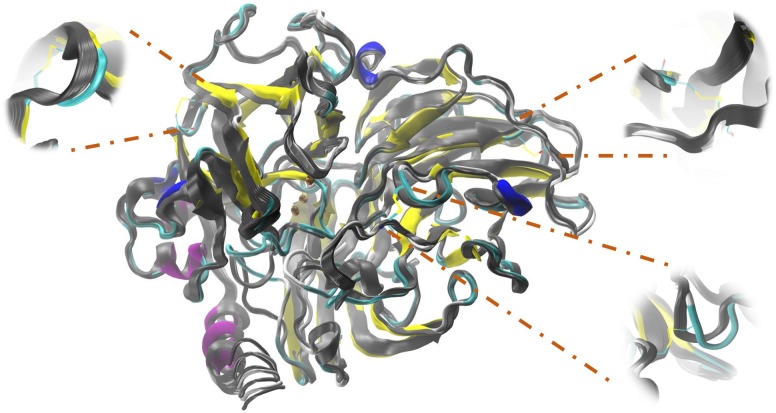
Simulation performed in ElNemo of low frequency movements for Lacc 6 and mutant 3-DB. An overlap of the 10 snapshots calculated within mode 7 of the mutated enzyme (in colors according to its secondary structure) on the native enzyme (gray scale), the low/high frequency of the structure indicated by the movement of the Cα which suffer a displacement of between 1.00 and 1.20 Å.

**FIGURE 5:**
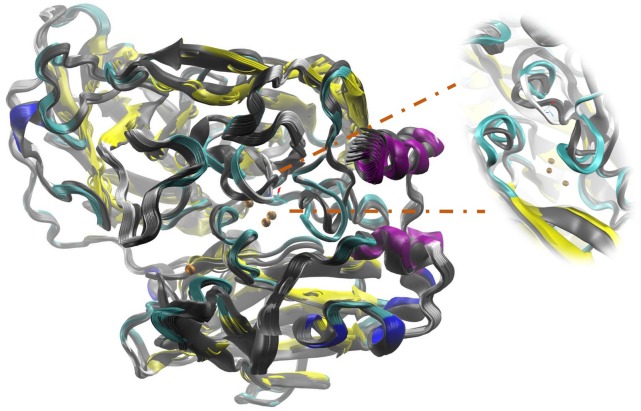
Structural overlap illustrating Active Site disturbances due to the mutation performed by replacing side chain Lysine 132 with a Threonine (L132T). The color image corresponds to the mutant enzyme and in grayscale to the native enzyme.

**FIGURE 6:**
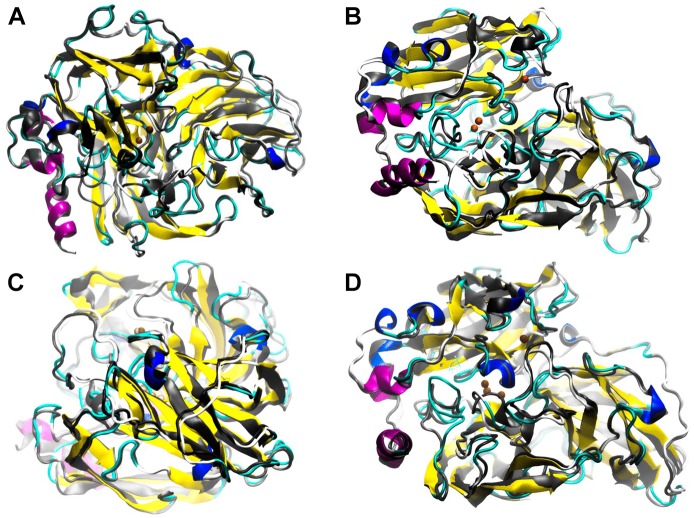
Low frequency motions corresponding to mode 7 calculated by ElNemo show a counter-rotation torsion between the domain for the distant copper and the binding domains to trinuclear site. In general, the perturbation to the global structure generated by the mutants: 3-SB **(A)**, L132T **(B)**, P203C **(C),** and F468P **(D)** is appreciated. ElNemo, overlapping of low energy motions on the minimized conformation between the native enzyme (grayscale) and the mutant (color coding according to secondary structure). These animations circulate around the 36 conformational snapshots, stored for low-frequency mode 7, centered on the minimized conformation of each type of enzyme. The regions of the most frequent proteins are highlighted given the counter-rotation movements between structural domains.

At a higher energy as shown in Table 4, but still moderate, with a change in the higher *R*^2^ for the 4-DB mutant, it exhibits vibrational movements and a flexibility more intense compared to Lacc 6, on the other hand, the 3-DB mutant also shows an even more moderate energy decrease than 4-DB, and also leads to a reduction in the change of R^2^, becoming a more rigid and less fluctuating structure as shown in Figure 4. The results show that for most of the mutants in which the change in the neighborhoods of the active site was made, it was disturbed as shown in Figure 5, where the L132T mutant is observed. These results indicate that most of the Lacc 6 regions and their mutants behave as a coupled structure that is mutually flexible or rigid, regardless of the energy calculated by the minimization. Inside the protein, displacement is close to 0. At the ends was observed a projection of displacement in the neighborhoods of the mutations; Left upper panel, D233C/T310C mutation; Right upper panel, K396C/D399C mutation and lower right panel, H184C/S284C mutation. On the mode 7, the native protein has greater variation compared to the mutant protein (Figure 6).

**FIGURE 7:**
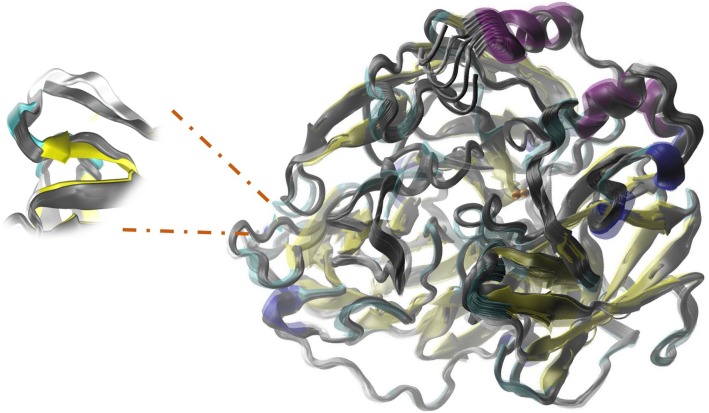
Relative movements in ElNemo mode7 between the mutant P203C and the native enzyme. The proteins are represented in the form of slats. The color-coded mutant depending on its secondary structure and native enzyme in grayscale. Also, it is shown an increase in fluctuation of native enzyme and the interconversion of non-random secondary structure for the formation of a beta-sheet on the opposite side to the mutation.

The three lower frequency movements, 7, 8, and 9, were selected as global representations of the movements (Table 5). Normal mode 7 was selected because values close to zero allow localized movements to be visualized, since normal low-frequency modes are expected to have collective characters, especially those related to the functional conformations of the protein (Tama and Sanejouand, 2001). In addition, as shown in Figure 7, the residues underwent significant displacement, although the structural deviation is small since the differences in RMSD (Root Mean Square Deviation) between the native protein and its mutants are about 1 Å, as can be seen in Table 5. Mode 7 shows a counter-rotation torsion between the structural domains, as can be seen in Figure 6, which would imply inclination and helical flexion, also one can observe that one of the domains rotate around an axis perpendicular to the plane of the membrane, while the others rotate around an inclined axis, this type of movements have not been described for this type of proteins.

**Table 5:** Root Mead Squarer Deviation (RMSD) values and displacement (fraction) for the mutant enzymes compared to Lacc 6.

Mutant	Mode	RMSD	Fraction of displacement
4-DB	7	1.097	0.3211
	8	1.109	0.0883
	9	1.109	0.5263
3-DB	7	1.004	0.0134
	8	1.004	0.5754
	9	1.004	0.5323
D233C-T310C	7	1.016	0.2418
	8	1.016	0.1575
	9	0.998	0.3829
F468P	7	1.004	0.4454
	8	1.004	0.0866
	9	0.999	0.5285
3-SB	7	1.014	0.1945
	8	1.014	0.1693
	9	1.011	0.5415
L132T	7	0.97	0.0456
	8	0.97	0.3889
	9	0.97	0.4427
N79D	7	1.044	0.0363
	8	1.044	0.4279
	9	1.034	0.4928
N372D	7	1.02	0.0716
	8	1.02	0.3694
	9	1.02	0.5128
P203C	7	0.966	0.055
	8	0.966	0.3882
	9	0.966	0.5439
P203V	7	1.028	0.0161
	8	1.028	0.5793
	9	1.028	0.5364
T147E	7	0.98	0.0597
	8	0.98	0.347
	9	0.98	0.5303
W85F	7	1.03	0.0271
	8	1.03	0.5306
	9	1.03	0.5073
Lacc 6	7		0.0794
	8		0.2892
	9		0.3396

*Column number 1 are the models of lower energy; in column 2, are the normal mode 7–9; in the column 3 the RMSD vs. Lacc 6; and the last column shows the fraction of residues that are significantly affected by a given mode.*

Flexibility and low energy motions were compared between the Lacc 6 and its mutants, ElNemo indicated that the low energy motions between the enzymes for their mutant and native form contain a nucleus in the protein that is immobile except for the L132T and F468P mutants (Figures 6B,D) for which the disturbance of the active site underwent significant alterations, in addition to some mobile external secondary structures.

**FIGURE 8:**
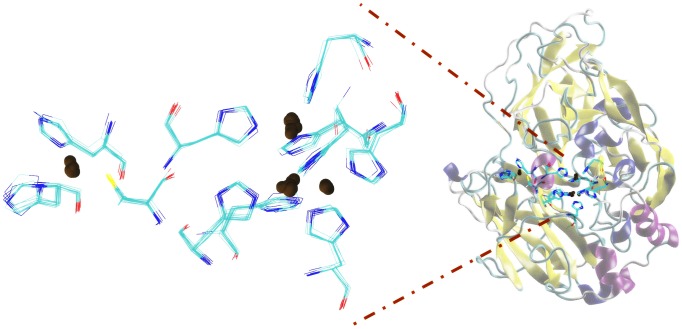
Domains close to the copper atom away from the trinuclear site of copper, showing the detail of the splicing of histidines bound to copper atoms.

Figures 4–7 make it possible to appreciate that the more flexible regions act as an interdomain hinge which allows coordinated movements of counter-rotational of each of these. ElNemo mode 7 shows the near domain to remote copper from the trinuclear copper site, it curves and straightens significantly according to the swinging of the contiguous domains, because the copper binding site implies a face of this domain (Figure 8 and Figure 1), these motions could be coupled to binding to the substrate or affect their affinity, especially in mutants designed to disturb the active site.

## Discussion

In this study, a bioinformatic analysis of enzyme Lacc 6 of *Pleurotus ostreatus* and minimization of energy of mutants of this same enzyme, to improve its stability in non-optimal conditions of activity for its possible synthesis and application in the degradation of agroindustrial residues that have been underutilized.

Agroindustrial wastes are a source of environmental pollution, since its composition is rich in lignin, cellulose and hemicellulose that require enzymatic complexes for its decomposition. For example, in 2006, Mexico produced about 76 million tons of agroindustrial waste from 20 types of cultivation and there are no physical, chemical, or enzymatic transformation techniques for their use (Valdez-Vazquez et al., 2010). Laccases have been associated with various biological functions, depending on the organism and its developmental conditions, are secreted into multiple isoforms and its most well-known and studied function is the degradation of lignin (Téllez-Téllez et al., 2008; Díaz et al., 2013).

On the other hand, these types of enzymes have applications of great interest in the industry and within their characteristics must be produced at low cost, be stable to the working conditions in wide ranges of pH and temperature for the treatment of pollutants (Téllez-Téllez et al., 2008; Miele et al., 2010; Díaz et al., 2013). In this study a proposal is made of an improved laccase that shows a minimum activation energy that allows it to be stable and active under adverse reaction conditions.

Several studies have been carried out on the generation of *Pleurotus ostreatus* laccase enzyme mutants as an alternative to be applied in the biodegradation of xenobiotic and/or recalcitrant compounds as reported by Miele et al. (2010), who randomly performed the generation of 2300 mutants of the POXA1b (Lacc 6) isoenzyme of *Pleurotus ostreatus* to develop biocatalysts from this enzyme, but no proposal has been made to know how the possible mutations occurred on the active site of this enzyme and to obtain models that would show the effect on the trinuclear copper region by modifying amino acids adjacent to the histidines bound to these metals as is done in this research.

On the other hand, Prins et al. (2015), observed the effect of mutations near the Cu 1 copper site of Streptomyces coelicolor A3 (2), to evaluate their biochemical characteristics against the native enzyme, obtaining three mutations (M298F, V290N, and V290A), the V290N mutant showed approximately double the activity, four to five times greater was the activity of the mutant M298F, however, the thermostability was decreased. In contrast to this study, where the proposed mutations (3-DB, 3-SB, P203C) allowed us to see the effect on the secondary structure of the protein; the changes occurring in the active site region and the energy minimization suggests an increase in the thermostability of the protein and that could tolerate higher to optimal temperatures for prolonged times. Such mutants may be candidates for *in vitro* expression, as shown in multiple works (Christensen and Kepp, 2013a; Ghosh et al., 2016), in which *in silico* studies allow the generation and identification of molecular bases in an expeditious manner which are difficult to quantify experimentally, and *in vitro* studies are feasibly reproduced by *in silico* studies (Zou et al., 2016), showing that computational tools are a primary aid in the structural study of proteins. Given the results, the production of mutant enzymes is suggested through genetic engineering to be used in bioremediation processes.

In recent years, computational design has been successfully applied for the thermostabilization of enzymes with potential use in biotechnological processes, mimicking the evolution in the laboratory to develop more stable enzyme variants and, more recently, using rational strategies of computer-assisted enzymatic engineering. Festa et al. (2008) performed one of the first notable works in the computational stabilization of a laccase, presented a screening library of 1100 clones of the mutant 1M9B obtained from *Pleurotus ostreatus*, and showed a single mutation (L112F) giving rise to a more active enzyme but less stable with respect to the native enzyme (POXA1b or Lacc 6), same enzyme used in our research.

On the other hand, Christensen and Kepp (2013a,b), exhaustively studied the isoforms of the laccase of *Tramentes versicolor* by molecular dynamics and other computational techniques in which they predict and rationalize a laccase to provide stability to mutations of multiple sites in its structure, showing that it is possible to generate stable mutants through computational studies, although to carry it out it turned out to be expensive. Ferrario et al. (2015), tested a thermal treatment with simulations of molecular dynamics on a laccase enzyme, observed that the enzyme conserved its geometry even making conformational changes in the labile parts. In contrast to our research in which through the protocol used to increase the thermal stability of Lacc 6 based on a rational approach by means of multiple methods including phylogenetic analysis, comparison with homologous proteins (particularly thermophilic), optimization of charged interactions (saline bridges and hydrogen bonds), optimization of waste and loops that show unfavorable Ramachandran angles as well as high B factors and computational design based on the structure, suggest a fast and inexpensive computational alternative.

## Conclusion

Normal modes indicate that the nucleus of the mutants is found without significant motions, however, the exposed sections and in particular the domain of the distant copper undergo movements counter-rotation and global compression. Likewise, it is predicted that lower energy mutants such as 3-DB, 3-SB, P203C minimize fluctuations by making the skeleton less flexible in the vicinity of them, decreasing the range of motions. In the same way it is found that the mutants L132T and D468P generate a conformational change in the vicinity of the active site. This work predicts the global movements of the enzyme laccase, while providing a new perspective of these enzymes. This allows us to discuss the character of the movement in multiple structures on the same basis and as expected the normal low frequency modes from the modeling of the elastic network provide a good description of the global movements of this enzyme, which allows us to understand the molecular basis of the structural stability of the Lacc 6 enzyme of *Pleurotus ostreatus* and its mutants. This suggests that it will have greater stability in its structure that will allow it to have oxidase activity at temperatures above the optimum.

## Author Contributions

RD performed the experimental design, directed the over-all study, and also drafted the manuscript. YM-F, GD-G and MA-R helped perform the analysis of results and revised the manuscript. LH-Z performed the protein modeling and revised the manuscript. All authors read and approved the final manuscript.

## Conflict of Interest Statement

The authors declare that the research was conducted in the absence of any commercial or financial relationships that could be construed as a potential conflict of interest.

## Footnotes

1 http://www.clustal.org/omega/

2 https://www.predictprotein.org/

3 https://blast.ncbi.nlm.nih.gov/Blast.cgi

4 https://salilab.org/modeller/

5 https://modbase.compbio.ucsf.edu/modweb/

6 http://dezyme.com/

7 http://www.rbvi.ucsf.edu/chimera

8 https://www.predictprotein.org/

9 http://www.clustal.org/omega/

10 http://weblogo.berkeley.edu/logo.cgi
